# Matrix-M Adjuvated Seasonal Virosomal Influenza Vaccine Induces Partial Protection in Mice and Ferrets against Avian H5 and H7 Challenge

**DOI:** 10.1371/journal.pone.0135723

**Published:** 2015-09-24

**Authors:** Freek Cox, Anna Roos, Nicole Hafkemeijer, Matthijs Baart, Jeroen Tolboom, Liesbeth Dekking, Koert Stittelaar, Jaap Goudsmit, Katarina Radošević, Eirikur Saeland

**Affiliations:** 1 Janssen Prevention Center, Center of Excellence of Janssen Research & Development, Pharmaceutical companies of Johnson and Johnson, Leiden, The Netherlands; 2 Viroclinics Biosciences, Rotterdam, The Netherlands; Icahn School of Medicine at Mount Sinai, UNITED STATES

## Abstract

There is a constant threat of zoonotic influenza viruses causing a pandemic outbreak in humans. It is virtually impossible to predict which virus strain will cause the next pandemic and it takes a considerable amount of time before a safe and effective vaccine will be available once a pandemic occurs. In addition, development of pandemic vaccines is hampered by the generally poor immunogenicity of avian influenza viruses in humans. An effective pre-pandemic vaccine is therefore required as a first line of defense. Broadening of the protective efficacy of current seasonal vaccines by adding an adjuvant may be a way to provide such first line of defense. Here we evaluate whether a seasonal trivalent virosomal vaccine (TVV) adjuvated with the saponin-based adjuvant Matrix-M (MM) can confer protection against avian influenza H5 and H7 virus strains in mice and ferrets. We demonstrate that mice were protected from death against challenges with H5N1 and H7N7, but that the protection was not complete as evidenced by severe clinical signs. In ferrets, protection against H7N9 was not observed. In contrast, reduced upper and lower respiratory tract viral loads and reduced lung pathology, was achieved in H5N1 challenged ferrets. Together these results suggest that, at least to some extent, Matrix-M adjuvated seasonal virosomal influenza vaccine can serve as an interim measure to decrease morbidity and mortality associated with a pandemic outbreak.

## Introduction

Influenza is a negative strand RNA virus that can be classified into influenza A, B and C viruses. While influenza B and C mainly infect humans, influenza A infects a broad range of hosts including humans, birds and pigs [1]. The influenza envelope contains two major glycoproteins, namely hemagglutinin (HA) and neuraminidase (NA). To date, 18 different HA subtypes and 11 different NA subtypes have been identified [2]. There is a constant threat of influenza A viruses crossing the species barrier and causing human pandemics such as the pandemic outbreaks of H1N1 in 1918, H2N2 in 1952, H3N2 in 1968 and again an H1N1 in 2009 [3, 4]. Currently, descendants of the pandemic H3N2 and H1N1 strains of 1968 and 2009, respectively, and two influenza B strains circulate among humans and cause seasonal influenza epidemics [5].

Other subtypes of influenza A such as H5 and H7 viruses are classified as potential pandemic threats. The highly pathogenic avian influenza (HPAI) H5N1 virus is circulating in poultry in several countries in South-East Asia and in Egypt. From 2003 up till 2013 633 confirmed cases of human H5N1 infection have been reported of which 377 people have died resulting in a case fatality rate of 60% [6]. The avian H7N9 influenza A virus caused an epidemic outbreak in humans in China early 2013. Of the first 133 confirmed human cases, 43 people died resulting in case fatality rate of 32% [7]. Although for both these viruses human- to-human transmission has not been confirmed, the risk remains that this may occur. While the current H7N9 strains today show limited ability to be transmissible via droplets and aerosols (to become airborne) [8], recent studies have shown that only 5 mutations are required for an H5N1 virus to become airborne [9, 10]. These results emphasize that pre-pandemic preparedness is essential to ward off these threats.

Vaccination is considered the best way to prevent influenza infection and associated morbidity and mortality. Seasonal influenza vaccines and most pre-pandemic candidate vaccines are based on HA and NA mainly with the aim to induce antibodies directed to the receptor binding site located on the globular head of the HA and to prevent the interaction with host cells, thereby blocking viral entry. The globular head of the HA is, in particular, highly variable [11, 12], and therefore these classical influenza vaccines are mainly effective against closely related strains. Pandemic vaccines are known to be of lower immunogenicity than seasonal influenza vaccine [13–15] and they require a high antigen dose formulation or the addition of an adjuvant to be effective [16–19]. Vaccine preparedness is essential for a pandemic outbreak and production of a strain specific pandemic vaccine in response to an outbreak may take too long. An alternative way may be to develop a vaccine that can protect against a broad range of potential pandemic strains to lower pandemic influenza related morbidity and mortality. Adjuvation of seasonal influenza vaccines has the potential to extend their protective efficacy to pandemic strains [20–22].

Matrix-M MM) is a potent saponin-based adjuvant that consists of a mixture of two purified and well characterized saponins (fraction-A and fraction-C) [23]. Compared to the original formulation that contained both fractions in a single particle [24], the individual particle formulation has been shown to exhibit both improved adjuvant activity and safety profile in humans [19]. In the current study we evaluate the immune potentiating properties of MM in combination with a virosomal trivalent seasonal vaccine (TVV). In addition, we investigate whether TVV+MM is able to induce protection against potentially pandemic H5 and H7 virus strains in mice and ferrets.

## Results

### Cross-reactive antibody responses are enhanced by Matrix-M-adjuvation

**Fig 1:**
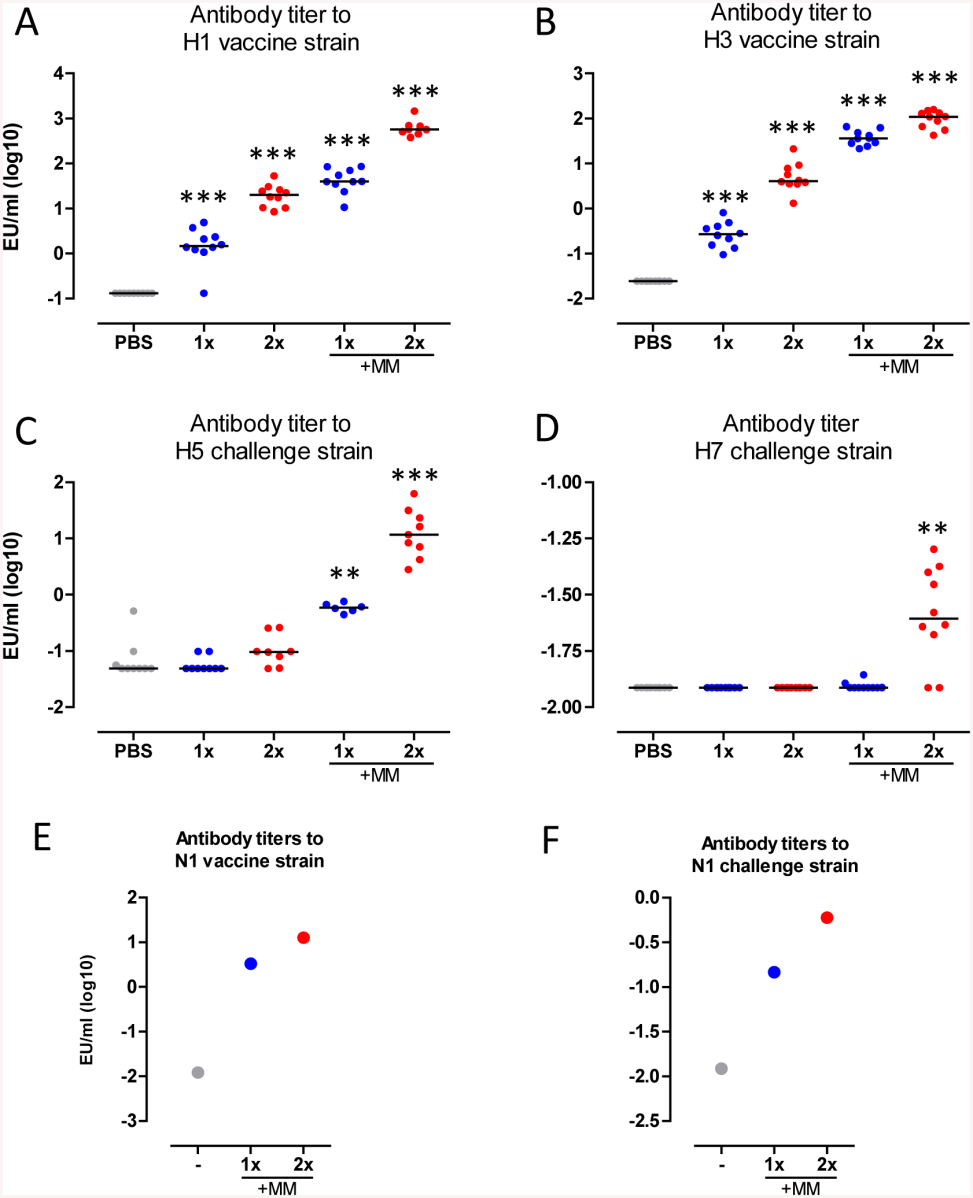
TVV+MM induces cross-reactive H5 and H7 antibody responses. Mice (n = 9-10/group) were immunized 1x or 2x with TVV with or without MM. 27 days later (1 day before challenge) individual serum samples were obtained and tested for (A) vaccine homologous recH1 of A/California/07/07, (B) vaccine homologous recH3 of the A/Victoria/210/09-like A/Perth/16/09 (98.8% homologous), (C) recH5 of A/Hong Kong/156/97, and (D) recH7 of A/Netherlands/219/03 (99.6% homologous to the challenge strain A/chicken/Netherlands/621557/03) antibody responses. Serum pools of mice (n = 50/group) that received 1x or 2x TVV+MM or no immunization (-) were tested for (E) vaccine homologous recN1 A/California/04/09 and (F) recN1 of A/Hong Kong/156/97 reactive antibody responses. Black bars indicate medians of log-10 transformed ELISA titers (EU). Asterisks indicate statistically significant differences compared to the vehicle control group (*p<0.05, **p<0.01, ***p<0.001, according to the materials and methods section).

To investigate the immune potentiating properties of MM, mice were immunized once or twice with TVV or TVV combined with MM (TVV+MM). Four weeks later individual serum samples were collected and vaccine homologous and challenge strain matching HA-specific antibody responses were determined. One and two immunizations with TVV alone yielded in statistically significant vaccine homologous H1 titers as compared to the vehicle control group (p<0.001 for both). The addition of MM increased the H1-specific antibody titers (p<0.001 for both compared to 1x and 2x TVV) (Fig 1A). A similar pattern of antibody responses were observed against vaccine homologous H3 demonstrating that immunizations with 1x or 2x TVV resulted in statistically significant induction of H3-spefific antibody titers as compared to the vehicle control group (PBS injected mice) (p<0.001 for both). The H3-specific antibody response was enhanced by adjuvation of TVV with MM (p<0.001 for both compared to 1x and 2x TVV) (Fig 1B). Next we assessed whether the improved vaccine homologous H1 and H3-specific antibody responses would translate in induction of cross-reactive antibody responses against the HA of avian H5 and H7 strains. Immunization with TVV alone did not result in significant levels of cross-reactive H5-specific antibody titers as compared to the vehicle control group. However, a single immunization with MM adjuvated TVV was sufficient to induce significantly higher antibody titers against H5 compared to the vehicle control group (p<0.001). A second immunization with TVV+MM further enhanced the H5-specific antibody response (p = 0.002 compared to 1xTVV+MM) (Fig 1C).

Two immunizations with TVV+MM were required to induce significantly higher cross-reactive antibody titers against H7 as compared to the vehicle control group (p = 0.001), while immunizations with TVV alone or 1x TVV+MM did not result in detectable H7-specific antibody responses (Fig 1D).

Next to the HA-specific antibody responses we also assessed the vaccine homologous and cross-reactive N1-specific antibody response in serum pools of mice immunized 1x or 2x with TVV+MM in comparison with pooled serum of naïve mice. Immunizations with TVV+MM resulted in vaccine homologous N1-specific antibody responses (Fig 1E). Interestingly, cross-reactive antibodies to the N1 of A/Hong Kong/156/97 (H5N1) were induced after immunizations with TVV+MM (Fig 1F).

### Matrix-M-adjuvated TVV protects mice against avian H5 and H7 influenza strains

Next, we investigated whether adjuvation of TVV with MM induces protection against avian H5 and H7 strains. Mice were challenged with a lethal dose of influenza virus A/Hong Kong/156/97 (H5N1) or A/Chicken/Netherlands/621557/03 (H7N7) four weeks after the final immunization and monitored for 21 days for survival, body weight and clinical scores.

**Fig 2:**
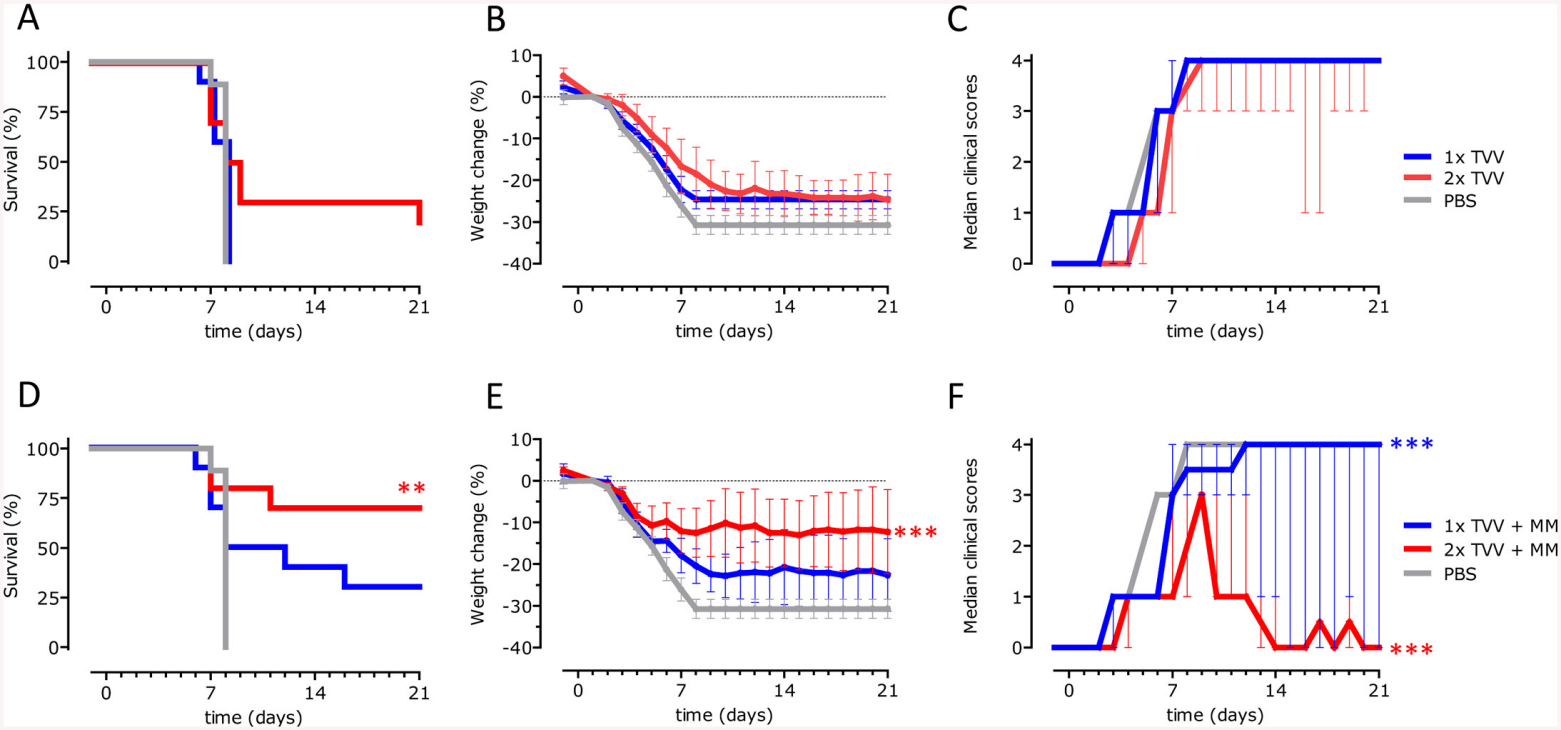
TVV+MM protects mice against avian H5N1. Mice (n = 9-10/group) were immunized 1x or 2x with TVV with or without MM. Four weeks later, mice were challenged with 25xLD_50_ wild-type A/Hong Kong /156/97 (H5N1) and monitored for 21 days for survival, body weight loss and clinical symptoms. Graphs represent the Kaplan-Meier survival curve (A and D) or mean bodyweight change with 95% confidence interval (B and E) or mean clinical scores with interquartile range (C and F). Asterisks indicate statistically significant differences compared to the vehicle control group (*p<0.05, **p<0.01, ***p<0.001, according to the materials and methods section).

After the lethal H5N1 challenge, all mice that received a single immunization with TVV alone succumbed to the infection, while a single immunization with TVV+MM partially protected the mice against death, although not significantly compared to PBS (p = 0.421). Two immunizations with the non-adjuvated vaccine did not improve survival proportions (p = 0.947 compared to PBS) whereas two immunizations with TVV+MM significantly increased survival proportions after H5N1 challenge compared to the vehicle control (p = 0.006) (Fig 2A and 2D). Accordingly, body weight-loss and clinical scores were significantly lower compared to PBS (p<0.001 for both) (Fig 2E and 2F).

**Fig 3:**
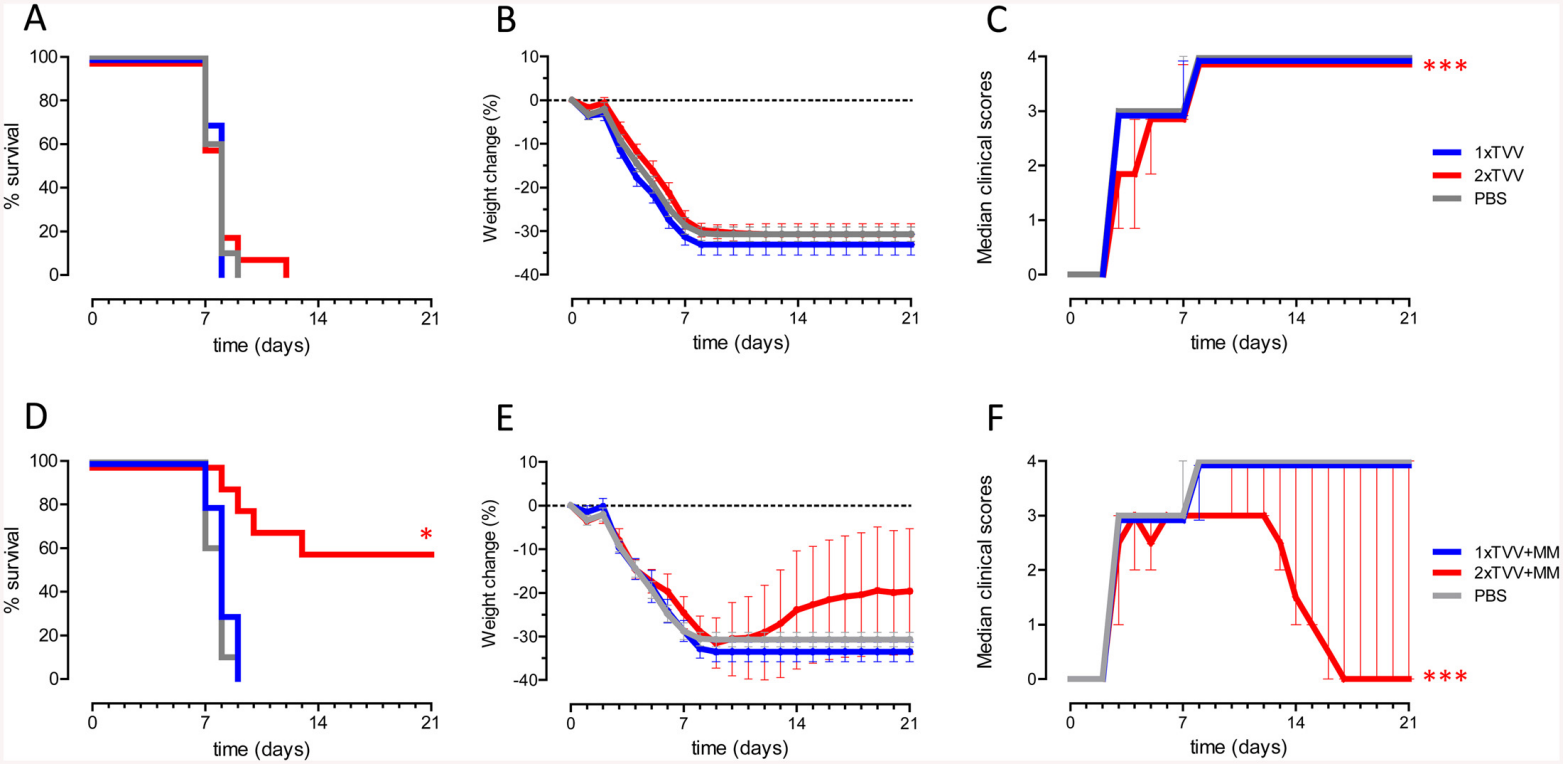
TVV+MM protects mice against avian H7N7. Mice (n = 10/group) were immunized 1x or 2x with TVV with or without MM. Four weeks later, mice were challenged with 25xLD_50_ H7N7 A/Chicken/Netherlands/62155710/03 and monitored for 21 days for survival, body weight loss and clinical symptoms. Graphs represent the Kaplan-Meier survival curve (A and D) or mean bodyweight change with 95% confidence interval (B and E) or mean clinical scores with interquartile range (C and F). Asterisks indicate statistically significant differences compared to the vehicle control group (*p<0.05, **p<0.01, ***p<0.001, according to the materials and methods section).

Next, we assessed whether TVV+MM was able to induce protective immunity against influenza virus H7N7. One or two immunizations with TVV or a single immunization with TVV+MM did not protect mice against death after H7N7 challenge (Fig 3A). However, two immunizations with TVV+MM resulted in 60% survival, reaching statistical significance compared to the vehicle control (p = 0.022) (Fig 3D). Comparable with the H5N1 results, protection against H7N7 was accompanied by substantial body weight loss and serious clinical signs (Fig 3E and 3F). While mice that received two immunizations with TVV+MM showed significantly reduced clinical signs (p<0.001), the body weight change did not significantly differ from the vehicle control group (Fig 3E and 3F).

To exclude that the observed protection in both models was induced by MM alone, mice were injected 3-times with 10μg MM alone with 3 week intervals and challenged with H5N1 or H7N7 4 weeks after the last administration. All mice succumbed to the lethal challenges (data not shown) indicating that MM enhances vaccine induced protection and does not induce a-specific protective immunity by itself.

### Matrix-M-adjuvated TVV partial protects ferrets against H5N1, but not against H7N9

Next we investigated whether protection against H5 and H7 strains in ferrets could be achieved by immunizations with TVV+MM. Ferrets were immunized twice with TVV or TVV+MM followed by sub-lethal challenge with either A/Indonesia/5/2005 (H5N1) or A/Anhui/1/2013 (H7N9). During an observation period of 4 days, body weight and body temperature were monitored and daily throat swabs were obtained to assess infectious virus titers. Four days after the challenge, ferrets were sacrificed and infectious lung viral titers and lung pathology were measured.

**Fig 4:**
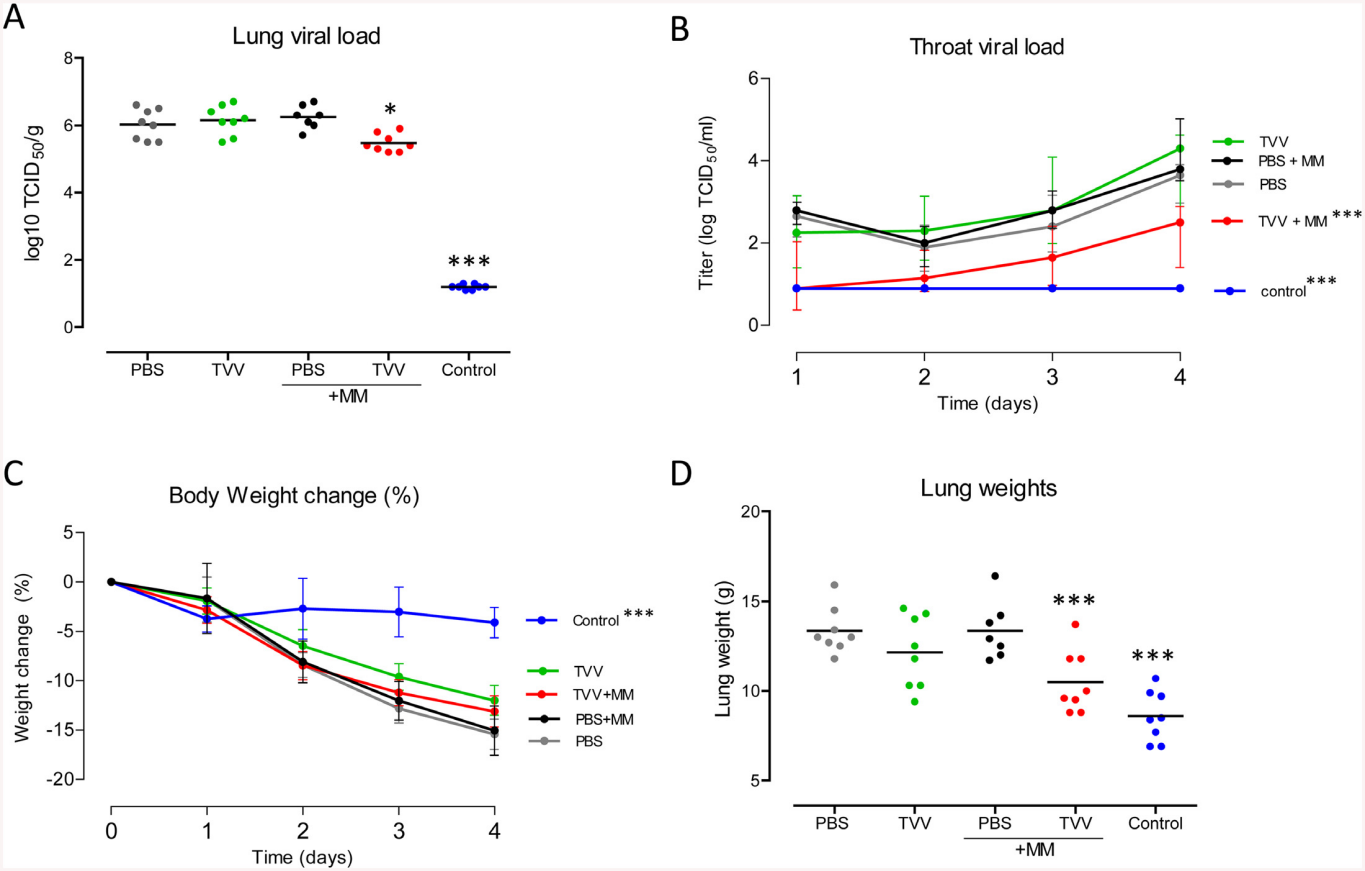
Ferrets are partially protected against highly pathogenic H5N1 after TVV+MM vaccination. Groups of 7–8 ferrets received two intramuscular injections with TVV, TVV+MM, PBS, PBS+MM or inactivated H5H1 virus as positive control (Control). 4 weeks later the animals were challenged with a sub-lethal dose of 10^4^ TCID_50_ of influenza A H5N1 A/Indonesia/05/2005. Ferrets were monitored for 4 consecutive days and sacrificed at day 4 post challenge. (A) Infectious viral load in lung tissue (B) infectious throat viral load (day 1 to 4), (C) percentage of body weight change during the observation period and (D) lung weight as determined after sacrifice. Dots indicate individual animals and horizontal lines represent group means (A and D). Lines represent group mean with 95% confidence interval (B) or the interquartile range (C). Asterisks indicate statistically significant differences compared to PBS injected animals (*p<0.05, **p<0.01, ***p<0.001, according to the materials and methods section).

Immunizations with TVV+MM resulted in a statistically significant 4.5-fold reduction in H5N1 infectious viral titers in the lung compared to animals injected with PBS only (p = 0.011) (Fig 4A). In addition, ferrets immunized with TVV+MM showed statistically significant reduced throat viral titers compared to the viral titers in the throats of animals receiving PBS (p<0.001) (Fig 4B).

Ferrets injected with TVV or MM alone did not show a statistically significant reduction of viral loads in the lung or throat compared to vehicle control, while control animals that were immunized with the homologous inactivated H5 virus showed a strong reduction in lung and throat viral titers (p<0.001 for both) (Fig 4A and 4B).

In agreement with the reduced viral load in the lungs of TVV+MM immunized animals, the mean lung weight (a measure of lung inflammation) in that group was significantly lower than the mean lung weight of PBS injected animals (p<0.001) (Fig 4D). These observations were in accordance with the observed lung lesions which were lower in the TVV+MM immunized animals compared to the PBS control animals (p = 0.002), although they did not significantly differ from animals injected with MM only (p = 0.262) (data not shown).

The reduced infectious viral load in the lungs and throat of TVV+MM immunized animals was not associated with reduced body weight loss (Fig 4C) or body temperature compared to the PBS control animals (S1A Fig), indicating that the immunized animals were not completely protected from disease during the observation period. TVV+MM immunized ferrets showed a statistically significant increase in body temperature across the observation period compared to the group receiving MM alone or PBS (p = 0.011 and 0.028 respectively), although they did not differ in body temperature compared to ferrets immunized with TVV alone (p = 0.548).

**Fig 5:**
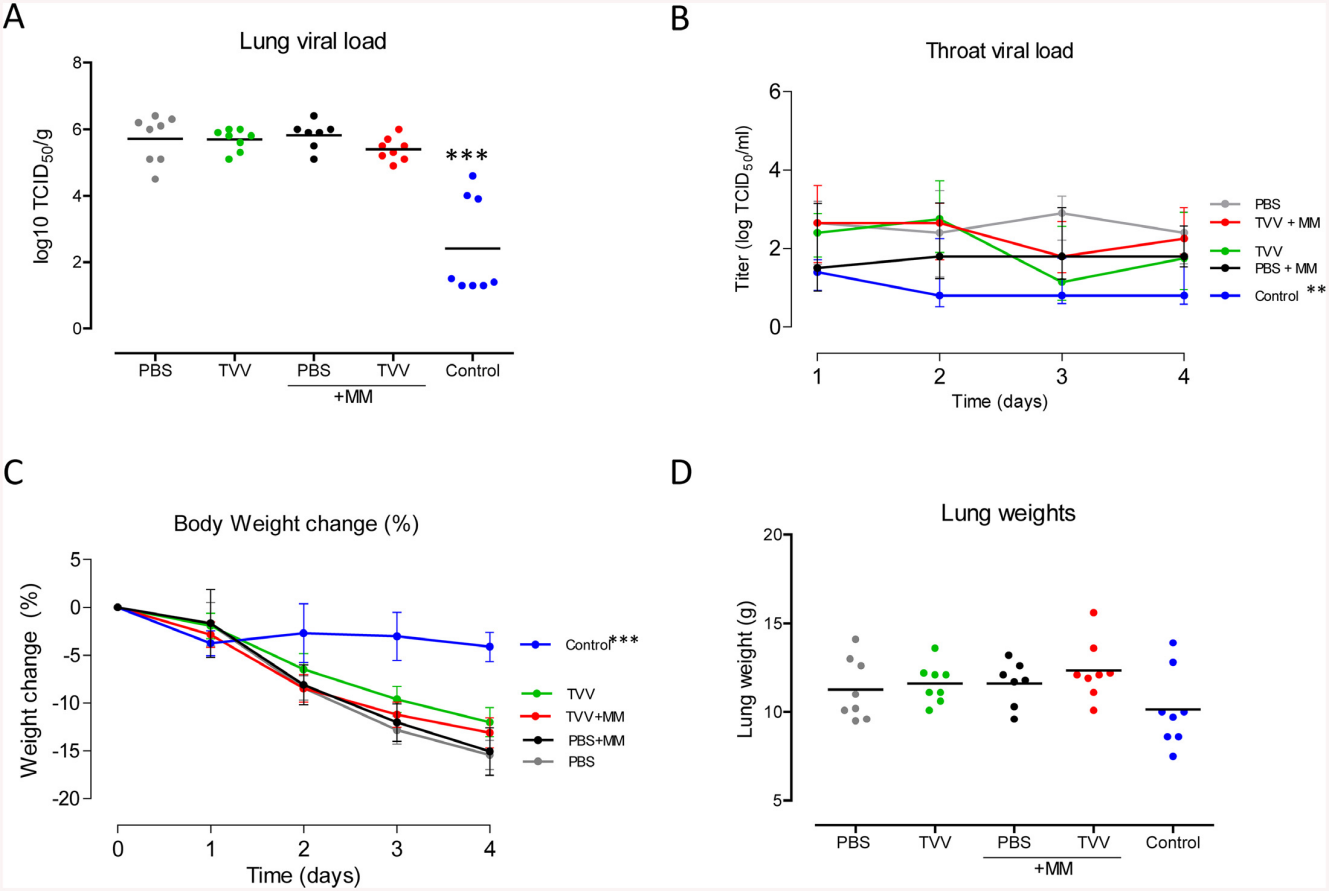
Ferrets are not protected against highly pathogenic H7N9 after TVV+MM vaccination. Groups of 7–8 ferrets received two intramuscular injections with TVV, TVV+MM, PBS, PBS+MM or inactivated H7N9 virus as positive control (Control). 4 weeks later the animals were challenged with a sub-lethal dose of 10^5.5^ TCID_50_ of influenza A H7N9 A/Anhui/1/2013. Ferrets were monitored for 4 consecutive days and sacrificed at day 4 post challenge. (A) Infectious viral load in lung tissue (B) infectious throat viral load (day 1 to 4), (C) percentage of body weight change during the observation period (D) lung weight as determined after sacrifice. Dots indicate individual animals and horizontal lines represent group means (A and D). Lines represent group mean with 95% confidence interval (B) or the interquartile range (C). Asterisks indicate statistically significant differences compared to PBS injected animals (*p<0.05, **p<0.01, ***p<0.001, according to the materials and methods section).

The protective effect observed in ferrets immunized with TVV+MM and challenged with H5N1, was not observed in TVV or TVV+MM immunized ferrets that were challenged with a sub-lethal dose of the H7N9 virus. In these animals, no significant reduction in viral load in the lung or throat was found. Accordingly, body weight change, body temperature, lung weights or lung lesions in animals immunized with TVV+MM did not differ from vehicle controls (Fig 5A–5D and S1B Fig).

## Discussion

Pandemic or epidemic outbreaks of highly pathogenic influenza A viruses have caused significant morbidity worldwide. To be prepared for such outbreaks it remains a high priority to develop vaccines that can protect against a broad range of influenza virus strains. In the current study we demonstrate that a virosomal trivalent seasonal vaccine adjuvated with the saponin-based adjuvant Matrix-M provides partial protection to mice in stringent challenge models with HPAI H5N1 and H7N7 strains. In ferrets TVV+MM significantly reduces viral load after H5N1 challenge. However, viral loads are not reduced in TVV+MM immunized ferrets that are challenged with the H7N9 strain.

The potential of MM in combination with a pandemic H5N1 virosomal influenza vaccine candidate has previously been evaluated in mice [25, 26] and humans [19, 27]. In both species, MM demonstrated a vaccine dose sparing effect, an advantage of an effective adjuvant. Furthermore, these studies showed that adjuvation with MM induced cross-reactive HAI responses against heterologous H5N1 where the non-adjuvated pandemic candidate vaccine failed [19, 25–27].

Here we show that adjuvation with MM enhances vaccine homologous and heterosubtypic HA and NA-specific antibody responses. In addition, a prime-boost administration of TVV+MM provides protection against avian H5N1 and H7N7 strains in mice. Immunizations with TVV+MM lowered the disease symptoms as evidenced by reduced clinical scores in both models and reduced weight-loss in the H5N1 challenge model as compared to the vehicle control group. However, disease symptoms in immunized mice were substantial. In agreement with these results, comparable heterosubtypic protection, that was accompanied by weight-loss, was observed in mice that received an experimental H1N1-based vaccine formulated as immune stimulating complexes (ISCOMs), also a saponin-based adjuvant system [20, 21].

Ferrets are considered a good animal model system to study influenza vaccine effectiveness against influenza because the α(2–6) sialic acid receptor distribution in the respiratory tract of humans and ferrets (the upper airway epithelia) has been shown to be comparable [28]. Furthermore, similar pattern of avian H5N1 and human H3N2 virus epithelial attachment has been demonstrated [29] and ferrets show human-like clinical signs such as sneezing, fever, and nasal discharge after influenza challenge [30]. Therefore we have evaluated the potential of TVV+MM to induce protection against potentially pandemic H5N1 and H7N9 virus strains also in a ferret challenge model. Ferrets were challenged intratracheally with sub-lethal challenge doses to allow comparison of lung pathology and lung viral loads. TVV+MM immunized ferrets were not protected against H7N9 virus challenge, but were partially protected against H5N1 virus as evidenced by both reduced lung- and throat viral loads and reduced lung weights compared to the vehicle control group. However, the TVV+MM immunized group of ferrets lost body weight similar to the PBS injected control group and had elevated body temperature compared to FI-H5N1 control group. In a different ferret model, Rockman et al. demonstrated that two immunizations with a split trivalent seasonal influenza vaccine (Fluarix composition of season 2005/06) adjuvated with alum or ISCOMATRIX protected ferrets from death and prevented severe clinical signs and body weight loss after intranasal administration of a lethal dose of H5N1 (A/Vietnam/1203/04). Vaccination had no significant impact on viral shedding as measured by nasal washings [22]. There are many variables between the two studies that may explain the contradictory observations such as the challenge strain and doses used, the vaccine composition, the adjuvants used and routes of administration. Despite the differences between the ferret models, in both studies the H5N1 infection with the H5N1 challenge viruses was not prevented, but partial protection was obtained. This indicates that seasonal influenza vaccines adjuvated with a saponin-based adjuvant can be used to reduce morbidity and mortality during a pandemic outbreak.

In order to improve influenza vaccines the understanding of the immunological parameters that mediate broad protection is crucial.

HA-specific antibodies that target the head-part of the HA and prevent the virus from attaching to the host cell are considered to mediate protection against vaccine matched strains. However, the homology between the vaccine strains and avian challenge strains are low and it is unlikely that head specific antibodies play a role in the observed protection. Recently, it has been shown that broadly neutralizing monoclonal antibodies that target the conserved stem region of the HA molecule confer protection in mice [31–35] and in ferrets [36]. The presence of broadly neutralizing stem-binding antibodies may explain the observed HA-specific cross-reactive antibody response and could (partially) explain the protection against avian H5 and H7 influenza strains in our study.

There is less focus on the role of antibodies against NA, however, NA-specific antibodies can also confer heterologous protection in mice and ferrets [22, 37–39] by preventing descendant viruses to egress and thereby inhibiting virus spread [40] or by mediating infected cell clearance via Antibody Dependent Cellular Cytotoxicity (ADCC) [41, 42]. It is conceivable that the cross-reactive N1-specific antibodies play a role in protection against H5N1. None of the NA subtypes in the vaccine match with the NA subtype of the H7 challenge strains (subtype N7 or N9), and this may explain why protection against the H7N9 strain was absent in the ferret model and less apparent in the H7N7 model in mice. The precise mechanism of protection against avian influenza strains induced by TVV+MM in mice is currently being evaluated.

In conclusion, Matrix-M adjuvation enhances the antibody response in mice and broadens the protective efficacy of a seasonal virosomal vaccine and provides protection against HPAI H5N1 and H7N7 influenza strains in mice and reduces viral loads in H5N1 challenged ferrets. Together, our data suggests that Matrix-M adjuvation can serve as an interim measure to decrease morbidity and mortality associated with pandemic outbreaks prior to the availability of matched vaccine.

## Materials and Methods

### Statement of Ethics

All mouse and ferret experiments were performed in accordance with Dutch legislation on animal experiments and approved by the DEC Consult (Independent ethical institutional review board).

### Mouse challenge studies

Six-to eight-week-old female BALB/c (H-2d) mice (specific pathogen-free) were purchased from Harlan (Boxmeer, The Netherlands) for the H5N1 study (performed at ViroClinics Biosciences, Rotterdam, The Netherlands) or from Charles River laboratories (Sulzfeld, Germany) for the H7N7 study (performed at Central Veterinary Institute (CVI), Lelystad, The Netherlands). The H1N1 A/California/07/09, H3N2 A/Victoria/210/09 and B/Brisbane/60/08 monovalent virosomes were prepared by Crucell (Berne, Switzerland) using conventional procedures [43]. In brief, the monovalent virosomes were mixed to obtain a trivalent virosomal vaccine (TVV) containing 3ug/100μl of HA per strain. Matrix-M (MM, 10μg/dose, Novavax AB, Uppsala, Sweden) was mixed with TVV just before immunization. For the H5N1 study, mice were anesthetized by isoflurane (IsoFlo, Abbott Park, IL, USA) while for the H7N7 study mice did not receive any anesthesia following 1x or 2x intramuscular (i.m.) injections with TVV, TVV+MM, MM only or PBS (total volume 100μl, 50μl per hind leg) 3 weeks apart. Blood samples were collected one day before injection and at one day before challenge (pre-challenge) 4 weeks after the last immunization to assess vaccine induced serum antibody responses. One day before H5N1 or H7N7 virus challenge, mice were placed in a BSL-3 isolator unit. Four weeks after the final immunization mice were anesthetized by intraperitoneal (i.p.) administration of 100mg/kg ketamine (Nimatek 100mg/ml, Eurovet, Cuijk, The Netherlands) in combination with 20mg/kg xylazine (Sedamun 20mg/ml, Eurovet) (CVI) or isoflurane (IsoFlo, Abbott Park) (Viroclinics). Mice were challenged with 25xLD_50_ H5N1 A/Hong Kong/156/97 (Viroclinics,) or H7N7 A/Chicken/Netherlands/621557/03 (CVI) via the intranasal route (a total of 50μl, 25μl per nostril).

Our experience with Influenza challenge models suggests that using alternative humane endpoints, such as bodyweight loss would lead to underestimation of protection. The amount of bodyweight loss before animals reach clinical score 4 is variable. Therefore we used clinical score 4 as a humane endpoint to accurately assess protection by vaccination. We explicitly discussed this issue with our Ethical Review Board, and they agreed to allow clinical score 4 as a humane endpoint.

After challenge, mice were monitored daily for weight loss, clinical score and survival for 21 days or until humane endpoint (clinical score 4). Clinical scores were defined as: 0 = no clinical signs, 1 = rough coat, 2 = rough coat, less reactive, passive during handling, 3 = rough coat, rolled up, labored breathing, passive during handling, 4 = rough coat, rolled up, labored breathing, unresponsive.

Although mice were monitored at least once a day to assess whether they reached the humane endpoint, we could not prevent that a large proportion of the mice died (44% in the H5N1 challenge and 99% in the H7H7 challenge model) as a consequence of influenza related illness in our very stringent H5 and H7 models. No gross pathology was performed to establish the exact cause of death. Mice that died before reaching humane endpoint were scored as clinical score 4. Mice that reached humane endpoint were sacrificed by cervical dislocation under isoflurane anesthesia as well as mice that were still alive at the end of the study.

### HA-and NA-based ELISA

To assess HA- and NA-specific binding antibody levels in serum samples, recombinant (rec) HA (Protein Sciences Inc., CT, USA) and recNA of H1N1 A/California/07/09 (produced on HEK293F cells) or recHA or recNA of H5N1 A/Hong Kong/156/97 (Both produced on HEK293F cells) or recH7 A/Netherlands/219/03 (Protein Sciences Inc.) were coated at a concentration of 0.5μg/ml onto Maxisorp 96-well plates (Nunc, Thermo Scientific) O/N at 4°C. Plates were washed with PBS (Life Technologies, Paisley, UK) containing 0.05% Tween-20 (Merck Millipore, Darmstadt, Germany) (PBS-T) and subsequently blocked with PBS containing 2% dried skimmed milk (Becton Dickinson, Breda, the Netherlands) for the HA-based ELISA or with PBS containing 2% bovine serum albumin (BSA; Sigma) (PBS/BSA) for the NA-based ELISA for 1 hour at RT. Following a wash with PBS-T, serum of individual mice (HA ELISA) or serum pools (NA ELISA) were added to the plate in duplicate, serially diluted (2- fold, 0.002–2%) and incubated for 1 hour at RT. Following a wash with PBS-T a 1:2000 dilution of goat-anti-Mouse HRP conjugated (KPL, Maryland, USA) was added to the plate and incubated for 1 hour at RT. After washing with PBS-T, OPD substrate (Thermo Scientific) was added to the plate. The colorimetric reaction was stopped after 10 minutes by adding 1M H2SO4. The optical density (OD) was measured at 492 nm and standard curves were created using a four parameter logistic curve. The OD of each sample dilution was then quantified against the standard (HA standard: human CR9114 [34] with a mouse IgG2a Fc-part, NA standard: mouse Monoclonal Antibody, clone 6G6 (Immune Technology Corp., New York, USA)) and the final concentration per sample (in ELISA Units, EU/ml) calculated by a weighted average, using the squared slope of the standard curve at the location of each quantification as weight. Negative samples were set at the limit of detection (LOD), defined as the lowest sample dilution multiplied by the lowest standard concentration with an OD response above the lower asymptote of the standard curve and background.

### Statistical analysis mouse challenge models

The serological data were analyzed by comparing between immunizations with TVV with or without Matrix-M relative to the vehicle control group receiving PBS. Data were log-transformed and comparisons between groups were made using the Wilcoxon rank-sum test with adjustment for multiple comparisons (2 fold Bonferroni for comparisons with PBS a stepwise approach testing first 2x and then 1x vaccination) (S1 Table). Additionally, the effect of two immunizations compared to one immunization and the effect of Matrix-M compared to TVV alone were determined. Data was log-transformed and comparisons between groups were made using the Wilcoxon rank-sum test with adjustment for multiple comparisons (4-fold Bonferroni).

For the challenges, the vaccine groups were compared to the vehicle control group for survival proportion, change in bodyweight and clinical scores. For survival proportion after challenge, a Fisher’s exact test was performed. For bodyweight loss and clinical score analysis, repeated measurements in the challenge phase were summarized as a single outcome per animal using an Area Under the Curve (AUC) approach where missing values for animals that died before day 21 were imputed with a last-observation-carried-forward method. Body weight data are expressed as the change relative to the day 0 measurement. The AUC was then defined as the summation of the area above and below the baseline. An ANOVA on AUC’s was done with group as explanatory factor. Clinical scores were summarized as AUC per mouse and groups were compared using a generalized linear model with a cumulative logit distribution to compare area under the curves for ordinal variable. The efficacy of the various treatment groups were compared to the vehicle treated control group using a Bonferroni adjustment for 2 comparisons (TVV +/- Matrix-M) followed by a step-wise approach (starting with the 2 immunizations and conditionally testing 1 immunizations if the previous step was statistically significant).

Statistical analyses were performed using SAS version 9.2 (SAS Institute Inc. Cary, NC, USA) and SPSS version 20 (SPSS Inc., IL, USA). Statistical tests were conducted two-sided at an overall significance level of α = 0.05.

### Ferret challenge studies

Seronegative (as determined by HI responses against circulating H1N1, H3N2 and B influenza strains as well as the challenge strains and antibodies against Aleutian disease virus) 12 month old outbred female ferrets (*Mustela putorius furo*) were obtained from Triple F (Sayre, PA, USA) and kept at the central animal facilities of Intravacc, Bilthoven, The Netherlands (standard housing)(Viroclinics). Ferrets received the TVV Inflexal V of season 2012/13 containing antigen of H1N1 A/California/07/09, H3N2 A/Victoria/361/11 and B/Massachusetts/2/12 (15μg/500μl HA per strain). Adjuvated TVV was obtained by adding 50μg of MM per dose just before immunization. Ferrets were immunized two-times i.m. with TVV or TVV+MM 3 weeks apart with 500μl vaccine (250μl per hind leg). Control groups received 500μl PBS or formaldehyde inactivated (FI) H5N1 or H7N9 challenge virus corresponding to 30μg total protein per dose formulated with 50μg MM by the same route. One day before H5N1 or H7N9 virus challenge, ferrets were placed in a BSL-3 isolator unit. Blood samples were collected at day 0 and 21 (pre-immunization) and at day 42 and day 49 (pre-challenge) to assess vaccine induced serum antibody responses (data not shown). Ferrets were anesthetized with 4–8mg/kg ketamine and 0.1mg/kg medetomidine before intratracheal inoculation with 10^4^ TCID_50_ A/Indonesia/5/05 or 10^5.5^ TCID_50_ A/Anhui/1/13 (H7N9) in 3 ml PBS. The challenge doses were based on studies showing substantial levels of virus replication in the upper and lower respiratory tract, as well as lung pathology, but no mortality during a 4-day follow-up period [44–46]. During the observation period of 4 days, body weight, body temperature and daily throat swabs were obtained to assess infectious virus titers. Temperature was recorded every 10 minutes by an interperitonally implanted temperature probe (DST milli-T logger; Star-Oddi, Reykjavik, Iceland). Four days after the challenge, ferrets were sacrificed by terminal bleeding under anesthesia with 4–8mg/kg ketamine and 0.1mg/kg medetomidine.

### Virus replication in upper and lower respiratory tract of ferrets

Nasal and throat swabs were taken daily during the 4-days observation period. After euthanasia samples of the right nasal turbinate and the right lung (four sections from each of the four (cranioventral, craniodorsal, caudoventral and caudodoral) lobes) were collected. Levels of viral RNA in the swab samples were determined by influenza A Matrix gene-specific TaqMan quantitative PCR (qPCR) [47]. Infectious virus load in qPCR positive swabs, nasal turbinates and lung tissue were determined by virus titration on Madin-Darby canine kidney (MDCK) cells [47]. Data were expressed as log TCID_50_ per gram of tissue or per ml in case of swabs.

### Ferret lung pathology

After sacrifice, the extent of pulmonary consolidation was assessed based on visual estimation of the percentage of affected lung tissue. Lungs were weighed and relative lung weight was calculated as proportion of the body weight on the day of sacrifice [18].

### Statistical analysis ferret challenge models

The TVV+MM group was compared to the PBS group for lung infectious virus titer (expressed as log^10^ TCID_50_/g) (primary outcome), the absolute and relative lung weight at day 4 of the challenge phase, the temperature recording, the daily measured infectious virus titer in throat swabs and the daily measured bodyweight.

Secondary comparisons were performed between TVV+MM and TVV to assess the added value of MM and between TVV+MM and MM alone and PBS and MM alone to assess effects of the adjuvant itself. Furthermore, TVV was compared to PBS to assess possible protective effects of non-adjuvated vaccine. The positive controls, FI-H5N1 or FI-H7N9 formulated with MM were compared to PBS group. An analysis-of-variance with treatment group as factor was performed followed by post-hoc t-tests. All comparisons were tested two-sided at the 5% significance level without adjustment for multiple comparisons.

Daily measurements were summarized per animal as AUC of the change relative to the day 0 measurement. The body temperature recording was summarized per animal as an AUC of the temperature change relative to the average in the challenge phase up to the actual challenge and an analysis-of-variance with treatment group as factor was applied to the (summarized) secondary outcomes. In the analysis-of-variance a transformation on the outcome values was used if it improved the normality of the data. Lung pathology, as scored by percentage of affected area that contained lesions, was analyzed with the Wilcoxon test.

Statistical analyses were performed using SAS version 9.4 (SAS Institute Inc. Cary, NC, USA) and SPSS version 20 (SPSS Inc., IL, USA).

## Supporting Information

S1 Fig Summary of temperature monitoring in H5N1 and H7N9 challenged ferrets. Groups of 7–8 ferrets received two immunizations with TVV, TVV+MM, PBS, PBS+MM or inactivated virus as positive control (Control) by the intramuscular route. Four weeks later animals were challenged with a sub-lethal dose of 104 TCID50 of influenza A H5N1 A/Indonesia/05/2005 or 105.5 TCID50 of influenza A H7N9 A/Anhui/1/2013. Temperature was measured every 10 minutes for 4 consecutive days and was summarized per animal as area under the curve (AUC) after H5N1 challenge (A) or H7N9 challenge (B). Dots indicate individual animals and horizontal lines represent group means. Lines indicate the 95% CI of the mean. Asterisks indicate statistically significant differences compared to PBS injected animals (*p<0.05, **p<0.01, ***p<0.001, according to the materials and methods section).
(TIFF)

S1 Table Summary of statistics analyses of H5N1 and H7N7 mouse challenge and antibody responses. Table summarizes *p*-values of survival proportion, body weight loss and clinical scores and the antibody responses of experimental groups as compared to vehicle control group (PBS) in the H5N1 and H7N7 mice challenge experiments. Statistical analysis was performed as described in the material and methods section. TVV = Trivalent Virosomal Vaccine. MM = Matrix-M.
(TIF)

S2 Table Summary of H5N1 and H7N9 ferret challenge statistics. Table summarizes *p*-values of all performed comparisons in the H5N1 and H7N9 ferret challenge studies. Statistical analysis was performed as described in the material and methods section. TVV = Trivalent Virosomal Vaccine. MM = Matrix-M. FI = Formaldehyde inactivated.
(TIF)
