# Supplementary figures and images for: Matrix-M Adjuvated Seasonal Virosomal Influenza Vaccine Induces Partial Protection in Mice and Ferrets against Avian H5 and H7 Challenge
Source: PLoS One. 2015 Sep 24;10(9):e0135723. doi: 10.1371/journal.pone.0135723 (PMC4581625; doi:10.1371/journal.pone.0135723)

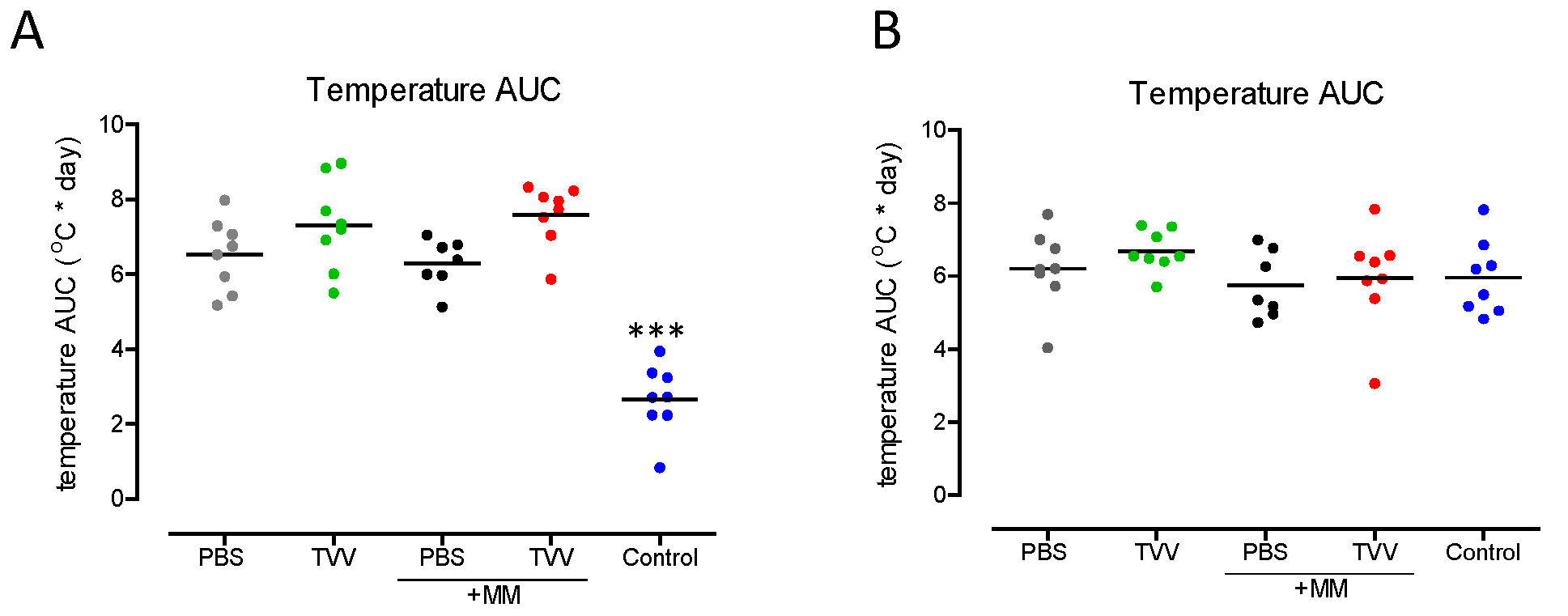

Supplement: S1 Fig — Groups of 7–8 ferrets received two immunizations with TVV, TVV+MM, PBS, PBS+MM or inactivated virus as positive control (Control) by the intramuscular route. Four weeks later animals were challenged with a sub-lethal dose of 104 TCID50 of influenza A H5N1 A/Indonesia/05/2005 or 105.5 TCID50 of influenza A H7N9 A/Anhui/1/2013. Temperature was measured every 10 minutes for 4 consecutive days and was summarized per animal as area under the curve (AUC) after H5N1 challenge (A) or H7N9 challenge (B). Dots indicate individual animals and horizontal lines represent group means. Lines indicate the 95% CI of the mean. Asterisks indicate statistically significant differences compared to PBS injected animals (*p<0.05, **p<0.01, ***p<0.001, according to the materials and methods section). (TIFF) [file pone.0135723.s001.tiff]

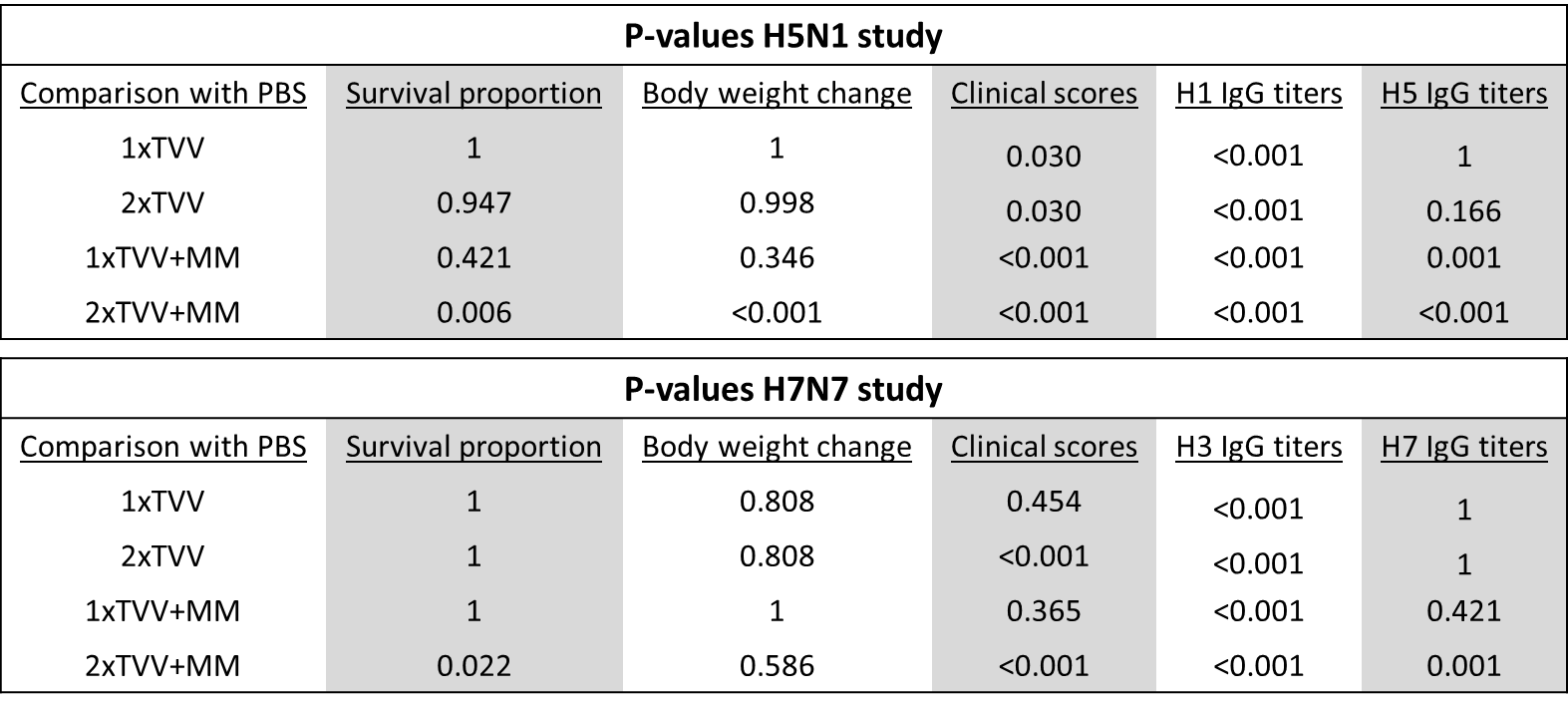

Supplement: S1 Table — Table summarizes p-values of survival proportion, body weight loss and clinical scores and the antibody responses of experimental groups as compared to vehicle control group (PBS) in the H5N1 and H7N7 mice challenge experiments. Statistical analysis was performed as described in the material and methods section. TVV = Trivalent Virosomal Vaccine. MM = Matrix-M. (TIF) [file pone.0135723.s002.tif]

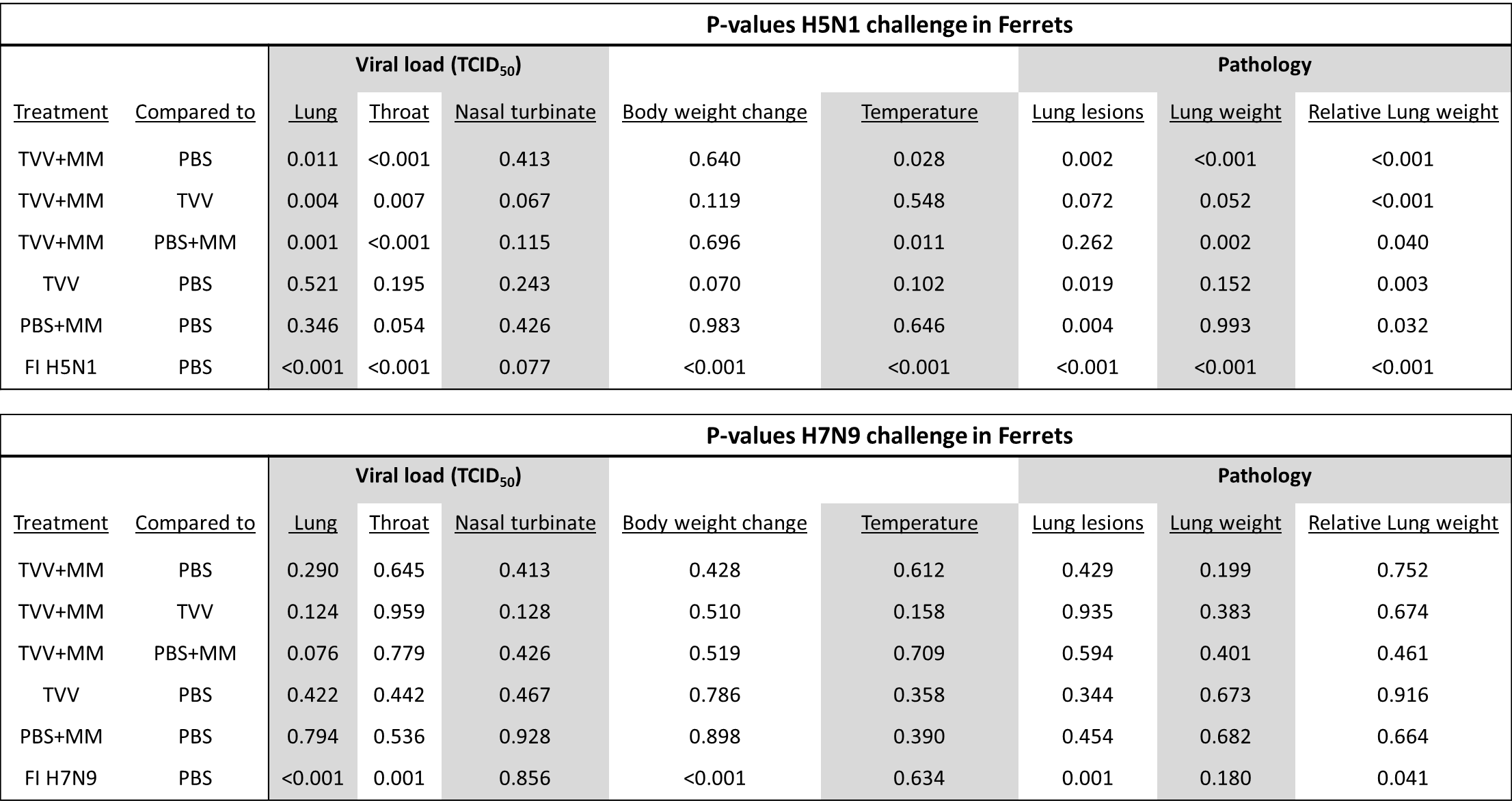

Supplement: S2 Table — Table summarizes p-values of all performed comparisons in the H5N1 and H7N9 ferret challenge studies. Statistical analysis was performed as described in the material and methods section. TVV = Trivalent Virosomal Vaccine. MM = Matrix-M. FI = Formaldehyde inactivated. (TIF) [file pone.0135723.s003.tif]
